# Preparation, Characterization, Swelling Potential, and In-Vitro Evaluation of Sodium Poly(Styrene Sulfonate)-Based Hydrogels for Controlled Delivery of Ketorolac Tromethamine

**DOI:** 10.3390/ph14040350

**Published:** 2021-04-09

**Authors:** Muhammad Suhail, Chih-Wun Fang, Muhammad Usman Minhas, Pao-Chu Wu

**Affiliations:** 1 School of Pharmacy, Kaohsiung Medical University, 100 Shih-Chuan 1st Road, Kaohsiung City 80708, Taiwan; Suhailpharmacist26@gmail.com; 2 Divison of Pharmacy, Zuoying Branch of Kaohsiung Armed Forces General Hospital. No. 553, Junxiao Rd., Zuoying Dist., Kaohsiung City 81342, Taiwan; cjs010801@hotmail.com; 3 College of Pharmacy, University of Sargodha, Sargodha 40100, Pakistan; 4 Department of Medical Research, Kaohsiung Medical University Hospital, Kaohsiung 80708, Taiwan; 5 Drug Development and Value Creation Research Center, Kaohsiung Medical University, Kaohsiung 80708, Taiwan

**Keywords:** hydrogels, sol-gel analysis, dynamic swelling and in-vitro percent drug release

## Abstract

The objective of the current study work was to fabricate sodium poly(styrene sulfonate-co-poly acrylic acid) (SPSPAA) hydrogels by using a free radical co-polymerization method for controlled delivery of ketorolac tromethamine (KT). Polymer (sodium poly(styrene sulfonate) (SPS) polymerized with monomer acrylic acid (AA) in the presence of initiator ammonium peroxodisulfate (APS) and cross-linker *N*′,*N*′-Methylene bisacrylamide (MBA). Different combinations of polymer, cross-linker and monomer, were employed for development of polymeric hydrogels. Various studies such as sol-gel, drug loading, dynamic swelling, and drug release studies were carried out to know the sol and gel portion of SPSPAA, swelling behavior of hydrogels at different pH media (1.2 and 7.4), quantification of drug loaded by fabricated hydrogels, and amount release of KT at pH 1.2 and 7.4. Higher dynamic swelling was found at pH 7.4 compared to pH 1.2, and as a result, greater percent release of drug was perceived at pH 7.4. Thermal stability, crystallinity, confirmation of functional groups and development of a new polymeric system, and surface morphology were evaluated via Thermogravimetric Analysis (TGA), Differential Scanning Calorimetry (DSC), Powder X-ray Diffraction (PXRD), Fourier Transform Infrared Spectroscopy (FTIR) and Scanning Electron Microscopy (SEM) respectively. The results showed that the present work could be used as a potential candidate for controlled delivery of KT.

## 1. Introduction

Non-steroidal anti-inflammatory drugs (NSAIDs) are the most commonly prescribed drugs to approximately 20% of patients above 65 years who are on NSAID medication [1]. Due to the higher analgesic potency and lesser side effects as compared to opioids, NSAIDs play an important role in management of acute pain such as headache, flu, stomachache, etc. Ketorolac tromethamine (KT) is a NSAID and used in relieving of severe pain with high analgesic and low anti-inflammatory activity [2,3]. Less hepatic first-pass elimination is shown by KT according to reported data. The biological half-life of KT is 2.5 to 4 h. Due to the short half-life, repeated dose administration is required to maintain a therapeutic level. Complications such as gastrointestinal ulceration and acute renal failure may be produced due to repeated administration of KT dose for a long period of time [4]. Frequent usage of KT reduces the patient compliance. Therefore, a suitable controlled drug delivery system is needed to overcome the complications and adverse effects associated with repeated doses of KT [5].

Hydrogels are three-dimensional cross-linked networks of polymer chains having the capability to absorb and hold a high quantity of water without losing their structural integrity [6,7]. The absorbed solution remains inside the swollen hydrogels even in the presence of any external pressure [8]. Due to the presence of a large number of hydrophilic groups such as –OH, –NH_2_, –SO_3_H, –COOH, etc. on the polymer chains, hydrogels absorb and hold a significant quantity of water [9]. Hydrogels play an important role in tissue engineering and drug delivery due to their unique properties such as their softness, superabsorbancy, hydrophilicity, viscoelasticity, biocompatibility, biodegradability, and their similarity with extra cellular matrix. Most importantly, hydrogels cause minor tissue damage or negligible toxicity and do not show any inflammatory responses or thrombosis. The reversible responses of hydrogels to various stimuli such as pH, temperature, magnetic field, electric field, ionic strength of solution and biological molecules, is another astonishing property [10] that further increases their importance, particularly for a wide range of biomedical applications [11]. 

Stimuli-sensitive hydrogels are the special type of hydrogels which are sensitive by nature to specific changes in the environment and show their responses by either changing their shape or volume once exposed to certain conditions. Recently, researchers have focused on pH-sensitive hydrogels as they are considered to be the most studied hydrogels amongst stimuli-sensitive hydrogels. The abrupt changes that take place when stimuli-responsive hydrogels are exposed to a specific stimulus leading to volume phase transition are their collapses and swelling. The size, shape, number of ionic groups, composition, and cross-linking density of hydrogels are the main factors which determine the responsive rate of hydrogels once exposed to a specific stimulus. The higher the pore size and number of ionic groups, the higher the response of hydrogels and vice versa [12].

Sodium polystyrene sulfonate (SPS) is the sodium salt of polystyrene sulfonate and has a variety of applications such as multilayer polyelectrolyte membranes for controlled drug delivery, medicines to treat hyperkalemia (abnormally high potassium or lithium levels in blood), cosmetics, and proton exchange membranes for fuel cells. It also plays an important role in the development of a polymeric system as it has the maximum swelling capability at a particular pH due to a high uptake of free ions. Due to the presence of different functional groups, especially SO_3_, SPS is used widely in biomedical and pharmaceutical fields. Acrylic acid (AA) is a water soluble polymer used mostly in stimuli-sensitive polymeric carrier systems, especially in pH-sensitive hydrogels. Due to its pH-sensitive nature, it has widespread biomedical and pharmaceutical applications as it delivers the maximum concentration of drug at a target site. Like other pH-sensitive polymers such as Carbopol, chondroitin sulfate, and polyvinyl alcohol, AA exhibits a maximum swelling index at basic medium due to deprotonation of functional groups (COOH) as compared to acidic medium and, therefore, more rapidly releases the drug at basic medium.

Here we report on sodium poly(styrene sulfonate-co-poly acrylic acid (SPSPAA) hydrogels for controlled delivery of KT. Various concentrations of polymer, monomer, and cross-linker were employed for the fabrication of developed hydrogels. Sol-gel studies were conducted to analyze the sol and gel fraction of the fabricated hydrogels. Similarly, dynamic swelling and dissolution studies were carried out at two different pH media, i.e., pH 1.2 and pH 7.4, and higher swelling and drug release was found at pH 7.4 for developed hydrogels. Besides this, characterizations such as via Thermogravimetric Analysis (TGA), Differential Scanning Calorimetry (DSC), X-ray Diffraction (XRD), Fourier Transform Infrared Spectroscopy (FTIR) and Scanning Electron Microscopy (SEM) were performed for SPSPAA hydrogels to know the various features of the developed hydrogels.

## 2. Results and Discussion

### 2.1. Sol-Gel Analysis

Sol-gel analysis is carried out to know the sol and gel fraction of SPSPAA hydrogel formulations as shown in Table 1. The gel fraction is increased as the composition of the polymer, cross-linker and monomer increases. The increase in gel fraction due to increase in SPS composition at a constant composition of AA and *N*′,*N*′-Methylene bisacrylamide MBA is because of greater availability of reactive sites for monomer contents. As the composition of SPS increases, the number of reactive sites increases, and in-turn, an increase in polymerization reaction occurs. Khalid and his coworkers developed CS-co-poly(AMPS)-based hydrogels and found that as the concentration of the polymer increases, the gel fraction increases [13], which further supports our studies. Similarly, at a constant composition of SPS and MBA, the gel fraction increases by enhancing the composition of AA. Due to availability of higher reactive sites, the polymerization reaction is increased as the composition of AA is increased and vice versa. Like polymer and monomer, increase in the gel fraction is observed as the composition of MBA increases. The increase in gel fraction is because of the higher bulk density of the hydrogel network. The cross-linking process is enhanced by increasing the MBA composition, which leads to a decrease in the pore size of hydrogels, and as a result, a hard and tight network of hydrogel is developed [14]. Contrary to the gel fraction, the sol fraction is decreased as the composition of polymer, monomer, and cross-linker increases because the sol fraction is inversely proportional to the SPSPAA hydrogel fraction [15].

### 2.2. Dynamic Swelling Studies

**Figure 1:**
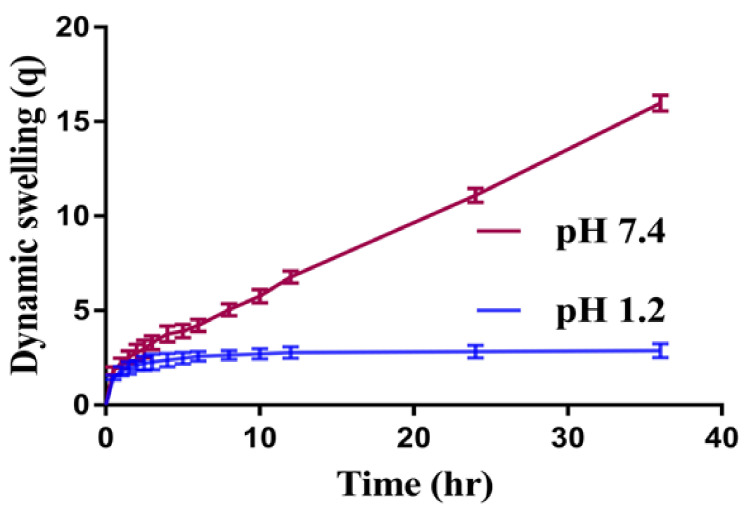
Effect of pH on dynamic swelling of SPSPAA hydrogel.

Dynamic swelling is performed to understand the swelling behavior of SPSPAA hydrogels at two different acidic and basic swelling media (pH 1.2 and pH 7.4), respectively, as shown in Figure 1. The pH value highly influences the dynamic swelling of SPSPAA as lower swelling is observed at pH 1.2 compared to pH 7.4. This behavior of hydrogels is because of the protonation of functional groups of SPS and AA at pH 1.2 and deprotonation at pH 7.4, respectively. The pKa value of SPS is −2.1 which means SPS is highly acidic by nature. Like 2-acrylamido-2-methyl propane sulphonic acid, SPS contains sulfonate groups. When SPS reacts with AA, it imparts pH-dependent behavior to the fabricated network of hydrogels. At lower pH 1.2, sulfonate ions of SPS are protonated and associated and give strength to the hydrogen bonding and thus generate a strong physical interaction among hydrogels. All these factors further generate additional strength and thus lead to a decrease in swelling at acidic medium [16]. Whereas at higher pH 7.4, sulfonate groups of SPS lead to ionization or deprotonation due to an increased charge density on the polymeric network, thus generating strong electrostatic repulsion among its ionized –SO_3_– groups, and higher swelling of SPSPAA hydrogels is exhibited [17]. These all lead to a decrease or loss of intermolecular hydrogen bonding, and thus, an increase in dynamic swelling is observed [18]. Similarly, at low pH 1.2, protonation of COOH groups of AA occurs, due to which the hydrogel network collapses and less swelling is exhibited. The pka of AA is about 4; hence, as the pH value of the medium increases from 6 to 8, deprotonation of COOH groups occurs, and as a result, electrostatic repulsive forces are increased among the COOH groups, and thus higher swelling is exhibited at higher pH 7.4 [19].

Similarly, the composition of polymer, monomer, and cross-linker also influences the dynamic swelling of SPSPAA gels at both acidic and basic media, as shown in Table 1. The dynamic swelling increases as the composition of SPS increases at constant composition of other contents. The possible reason is the increase of charge density on the polymeric system due to an increase in sulfonate ions which produce strong electrostatic repulsive forces, and hence, higher dynamic swelling is observed [20]. Similarly, increase in dynamic swelling is observed as the composition of AA increases while keeping the composition of SPS and MBA constant. Contrary to SPS and AA, decrease in dynamic swelling is shown by increasing the composition of MBA. A hard and tight junction is formed as MBA composition increases. The possible reason is the higher cross-linked bulk density that leads to reduction in the pore size of the hydrogel network, and thus, a decrease in dynamic swelling is detected [21,22].

### 2.3. Drug Loading

Drug loading was carried out for all formulations of SPSPAA hydrogels to know the quantification of the loaded drug by hydrogels as shown in Table 1. Drug loading is directly proportional to the swelling of hydrogels. The higher the swelling, the greater will be the drug loading [23] and vice versa. As the composition of SPS and AA increases, increase in the swelling of hydrogels occurs, and as a result, a greater amount of drug is encapsulated by the hydrogel contents [24]. Whereas in the case of MBA, a decrease in swelling is observed as the composition of MBA increases due to a higher cross-linking/bulk density, and thus, a decrease in drug loading is observed as the MBA composition is increased [23] and vice versa.

### 2.4. In-Vitro Drug Release and Kinetic Modeling

**Figure 2:**
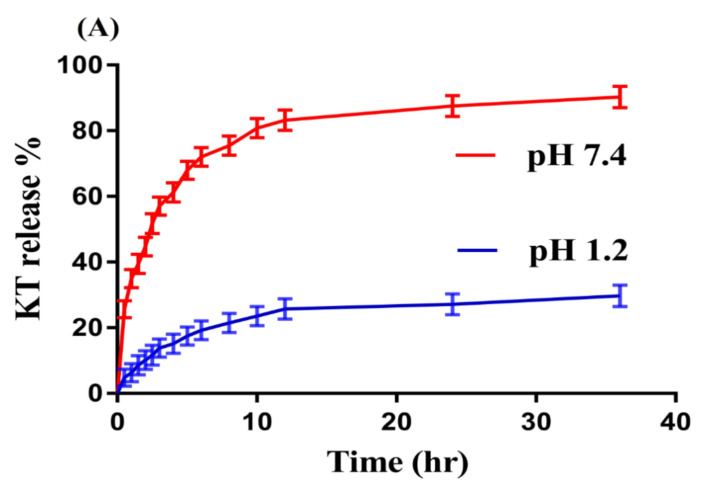

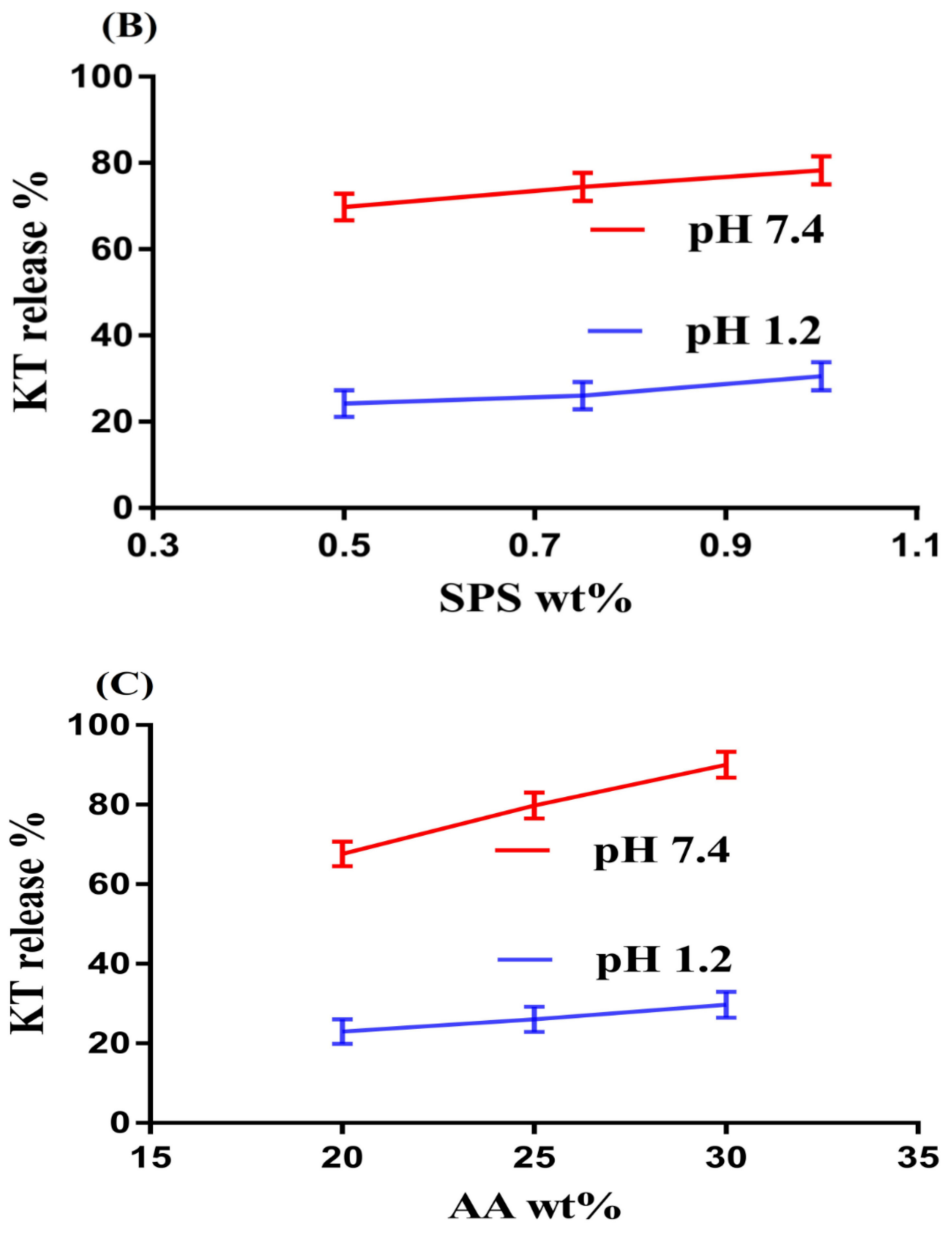

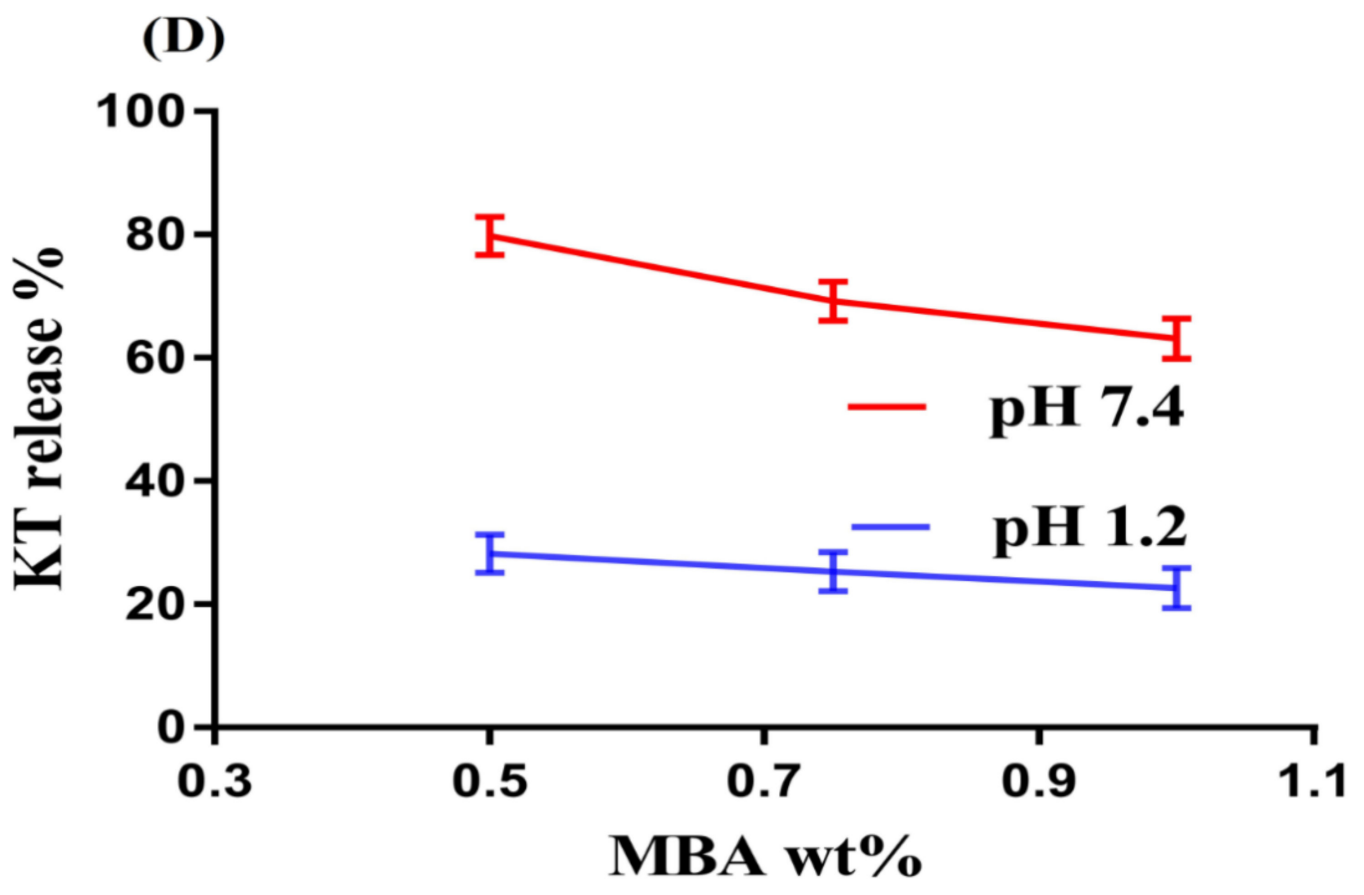
Effects of (**A**) pH, (**B**) SPS, (**C**) AA, and (**D**) *N*′,*N*′-Methylene bisacrylamide MBA on ketorolac tromethamine (KT) percent release from SPSPAA hydrogels.

An in-vitro drug release study was carried out for developed hydrogels at both low and higher pH (1.2 and 7.4) respectively as shown in Figure 2A–D. The result shows that pH highly influences the in-vitro drug release from developed hydrogels as a higher percent drug release is observed at pH 7.4 as compared to pH 1.2 (Figure 2A). The low percent drug release from SPSPAA hydrogels at pH 1.2 is due to the protonation of functional groups of SPS and AA that gives further strength to the hydrogen bonding. This all leads to low swelling, drug loading, and release of drug at pH 1.2. The greater percent release of drug from SPSPAA hydrogels at higher pH 7.4 is due to deprotonation of functional groups of SPS and AA that ionizes and enhances the charge density, and as a result, higher electrostatic repulsive forces are produced, which leads to higher swelling, loading, and release of drug from the fabricated polymeric system [17,25].

Like pH, the composition of hydrogels content also influences the release of drug from the fabricated network of hydrogels at both media, i.e., pH 1.2 and 7.4, respectively, as shown in Figure 2B–D. Increase in the percent drug release is observed as the composition of SPS increases at a constant composition of AA and MBA (Figure 2B). The increase in percent drug release is due to an increase in –SO_3_– groups that ionize highly as the composition of SPS increases and as a result the percent drug release is increased. Similarly, percent drug release increases as the composition of AA increases due to an increase in COOH groups (Figure 2C) [26] and vice versa. Unlike SPS and AA, a decrease in percent drug release is observed as the composition of MBA increases due to higher cross-linking and bulk density (Figure 2D) [27,28].

Kinetic models were applied for release data in order to know the mechanism of drug release from the developed polymeric system. The Korsmeyer‒Peppas model is considered the best kinetic model for SPSPAA hydrogels as compared to others kinetic models because “r” values of the Korsmeyer‒Peppas model are found higher than other kinetic models. The “r” values for the Korsmeyer‒Peppas model are found within the range of 0.8832–0.9829 (Table 2), which indicates that all formulations of fabricated hydrogels follow the Korsmeyer‒Peppas model. “n” values determine the type of diffusion, i.e., Fickian diffusion mechanism (*n* = 0.5) and non-Fickian or anomalous (*n* > 0.5) [29]. “*n*” values are found within the range of 0.3396–0.5299 (Table 2), which means that all formulations of developed hydrogels exhibit Fickian diffusion, except PSAF-4, which exhibits a non-Fickian diffusion mechanism.

### 2.5. TGA Analysis

**Figure 3:**
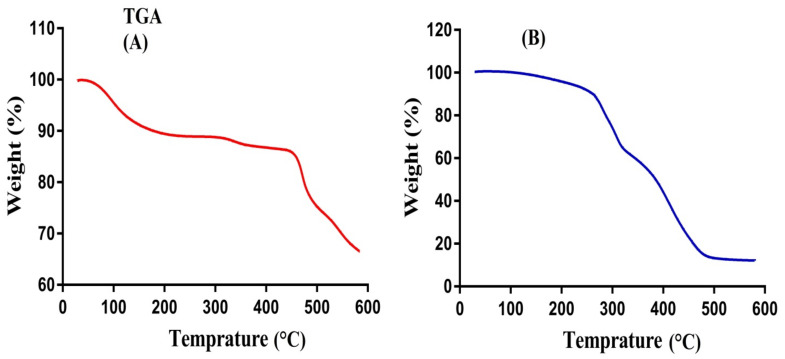
Thermogravimetric Analysis (TGA) of (**A**) SPS, (**B**) SPSPAA hydrogel.

The thermal stability of pure polymer SPS and SPSPAA hydrogels is observed by conducting TGA of a sample as shown in Figure 3A,B. Initially, TGA of SPS is carried out (Figure 3A). An initial weight loss of 10% is observed at a temperature range of 50–250 °C. After that, 7% weight reduction is detected within the range of 320–420 °C. Further increase in temperature leads to degradation of the polymer that starts from 430 °C until complete paralysis [30]. Thereafter, SPSPAA hydrogel is also subjected to TGA analysis (Figure 3B) and results are compared with pure polymer. The initial weight loss of 35% of the developed hydrogel is depicted by the TGA analysis at 208–280 °C due to loss of moisture contents. Similarly, a further weight loss of 20% is observed at 360–380 °C, and finally, 17% weight loss of fabricated hydrogel is found at 385–480 °C. After that, degradation of SPSPAA hydrogels starts until entire degradation. The degradation half-life of polymer is (t_1/2_ = 430 °C) whereas the degradation half-life of SPSPAA hydrogels is (t_1/2_ = 480 °C), which indicates that the developed system is thermally more stable then unreacted pure polymer and attributed to strong cross-linking between SPS and AA. Barkat and his co-workers prepared PEG 4000-based hydrogels and found higher thermal stability of the fabricated network compared to pure polymer [31].

### 2.6. DSC Analysis

**Figure 4:**
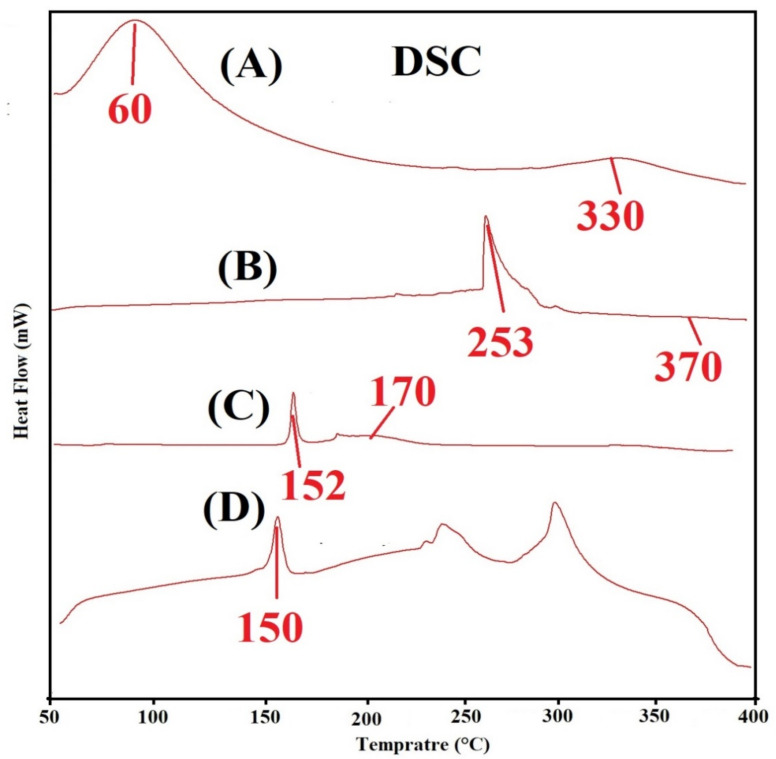
Differential Scanning Calorimetry **(**DSC) of (**A**) SPS, (**B**) SPSPAA hydrogel, (**C**) KT, and (**D**) loaded SPSPAA hydrogels.

The thermal stability of SPS, SPSPAA hydrogel, KT, and loaded SPSPAA hydrogels is conducted using DSC samples as shown in Figure 4A–D. Two endothermic peaks of SPS are detected at 60 and 330 °C (Figure 4A), respectively. The first broad endothermic peak represents the glass transition temperature while the later indicates the thermal degradation of SPS. Similarly, two endothermic peaks are observed via DSC analysis of SPSPAA hydrogels (Figure 4B). An endothermic peak at 253 °C is the peak of SPS that modifies from 60 to 253 °C in SPSPAA, whereas an endothermic peak of SPS at 330 °C disappears in the DSC thermogram of SPSPAA hydrogels due to a polymerization reaction of SPS with AA. The thermal degradation of the developed network starts at 370 °C, which indicates that the thermal stability of the SPSPAA hydrogel is higher than unreacted polymer. Barkat et al. prepared chondroitin sulfate-based hydrogels and reported higher thermal stability for the developed system compared to polymer [32]. Figure 4C indicates the DSC thermogram of KT. An endothermic peak at 152 °C is observed which reveals the melting point of the drug, whereas a minor endothermic peak is observed at 170 °C which is related to decomposition of the drug. The DSC thermogram of the loaded SPSPAA hydrogels is shown in Figure 4D. An endothermic peak is observed at 150 °C, which is the peak of drug. The slight change in peak position is due to the encapsulation of the drug by SPSPAA hydrogels. This all indicate that there is no interaction between the drug and contents of the hydrogels.

### 2.7. Powder X-ray Diffraction (PXRD) Analysis

**Figure 5:**
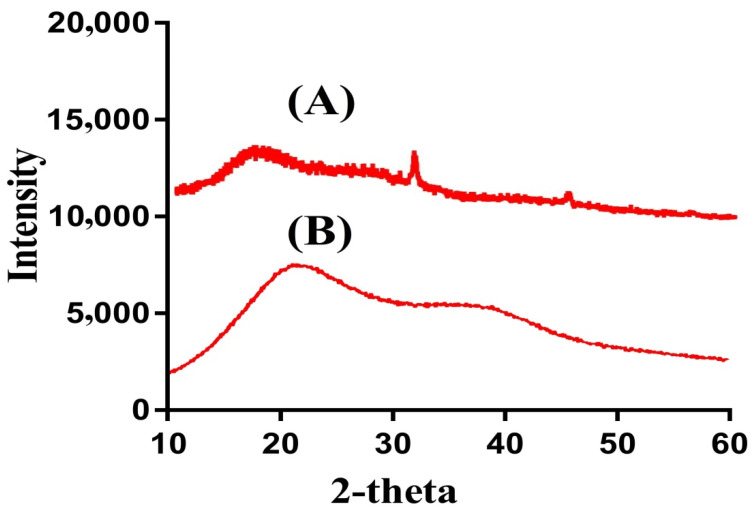
Powder X-ray diffraction (PXRD) of (**A**) SPS, (**B**) SPSPAA hydrogel.

PXRD is carried out for SPS and SPSPAA hydrogels as shown in Figure 5A,B. The PXRD diffraction pattern of SPS indicates two major diffraction peaks at angles 32.40° and 43.12° (Figure 5A), which can be attributed to the crystalline nature of SPS. The PXRD diffraction pattern of SPSPAA hydrogels shows a less crystalline nature as the intensity of two major peaks of the SPS is decreased in the developed hydrogels, as shown in Figure 5B. The decrease in crystallinity is because of the chemical bond formation between SPS and AA during the polymerization reaction. Lee and his coworkers synthesized amphiphilic poly(l-lactide)-grafted chondroitin sulfate copolymer hydrogel and found a reduction in crystallinity of individual ingredients in the X-ray pattern of polymerized network of hydrogels, which supports our observation in the current study [33]. 

### 2.8. Fourier Transform Infrared Spectroscopy (FTIR)

**Figure 6:**
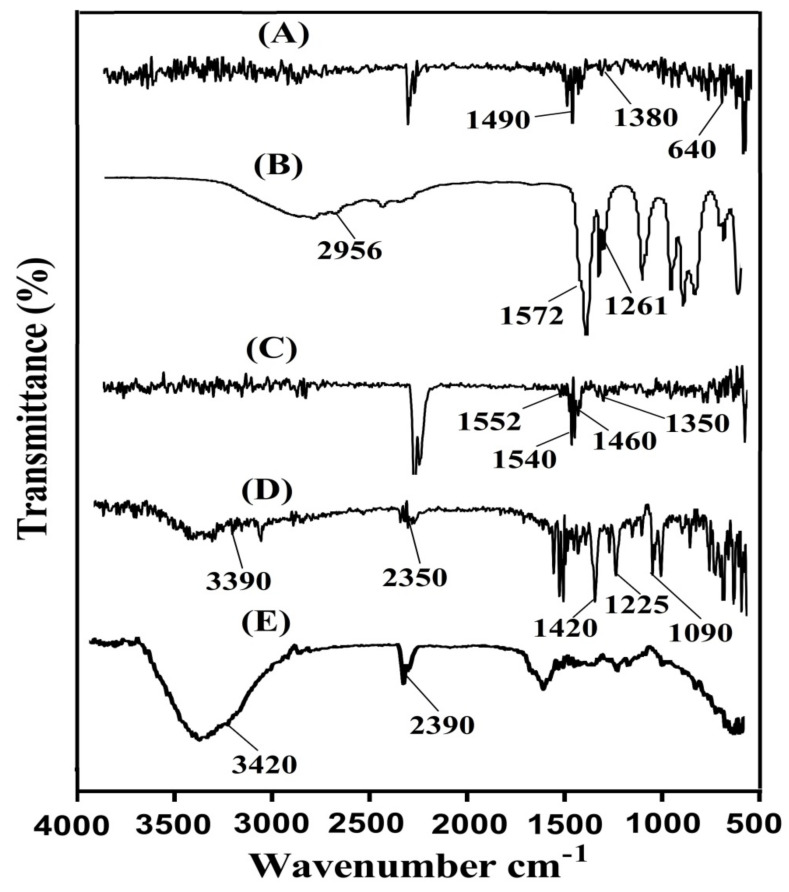
Fourier transform infrared spectroscopy (FTIR) of (**A**) SPS, (**B**) AA, (**C**) unloaded SPSPAA hydrogels, (**D**) KT, (**E**) loaded SPSPAA hydrogels.

FTIR spectra are analyzed for SPS, AA, KT, unloaded SPSPAA hydrogels, and drug-loaded SPSPAA hydrogels as indicated in Figure 6A–E, correspondingly. SPS (Figure 6A) shows FTIR spectra peaks at 1380 and 1490 cm^−1^ indicating the symmetric and asymmetric vibration of the SO_3_− group respectively. A peak at 640 cm^−1^ indicates aromatic C-H [34]. Similarly, distinct peaks of AA (Figure 6B) is assigned to the stretching vibration of –C–C and –CH_2_ at 1572 and 2956 cm^−1^, whereas the prominent peak at 1261 cm^−1^ is assigned to the stretching vibration of –C=O respectively [35]. The FTIR spectra of unloaded SPSCPAA hydrogels (Figure 6C) indicate a change in position of peaks for various functional groups of SPS and AA due to electrostatic interactions between them. The prominent peaks of SPS at 1380 and 1490 cm^−1^ are modified to 1460 and 1540 cm^−1^ peaks of unloaded SPSPAA hydrogels. Similarly, characteristic peaks of AA at 1572 and 1261 cm^−1^ are shifted to 1552 and 1350 cm^−1^, respectively. The disappearance of some peaks and formation of new peaks indicate the change in the intensity of the SPS and AA peaks. The above discussion indicates the development of SPSPAA hydrogels due to the cross-linking of SPS with AA on their reactive sites. The FTIR spectrum of KT (Figure 6D) exhibits peaks at 3390 cm^−1^ due to the stretching vibration of N-H and NH_2_, while the peak at 1420 cm^−1^ is due to –C–N vibrations. Similarly, two peaks at 1225 and 1090 cm^−1^ indicate the stretching vibration of C=O (diaryl ketone) and bending of –OH, which confirms the presence of an alcoholic group. Two peaks at 2190 and 752 cm^−1^ confirm the bending of C–H (aromatic) [36,37,38]. As indicated in Figure 6E, the FTIR spectra of drug-loaded SPSPAA hydrogels indicate a slight change in the prominent peaks of the drug due to encapsulation of the drug by the polymeric system. The characteristic peaks of the drug at 3390 and 2350 cm^−1^ are modified slightly to 3420 and 2390 cm^−1^ in loaded SPSCPAA hydrogels. The results demonstrated that the drug is encapsulated by SPSPAA hydrogels and no interaction of the drug with the contents of the hydrogels is observed [39].

### 2.9. Scanning Electron Microscopy (SEM)

**Figure 7:**
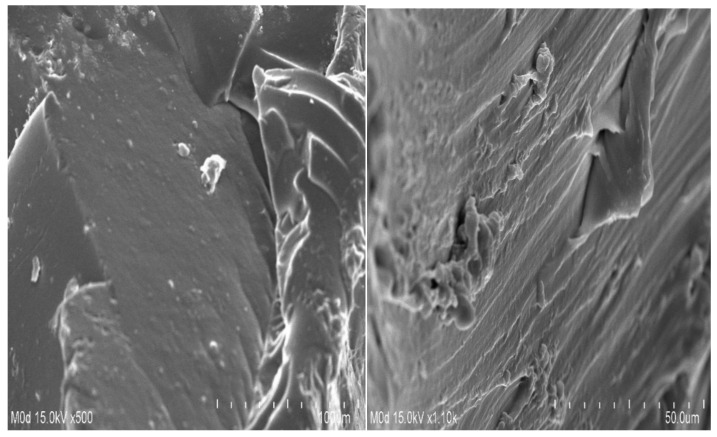
Surface morphology of SPSPAA hydrogels.

Scanning electron microscopy (SEM) is performed for the SPSPAA polymeric network to know its surface morphology. A rough surface with few pores is seen at different magnifications, as shown in Figure 7. The water penetrates through the pores into the hydrogel network, and as a result, the hydrogels swell. Drug loading depends on the swelling index of hydrogels. The higher the swelling, the greater the drug loading will be and vice versa. The rough surface of the developed hydrogels is because of good compatibility between the polymer, monomer, and cross-linker.

## 3. Materials and Methods

### 3.1. Materials

Ketorolac tromethamine (KT) was purchased from Symed Lab Limited (Telangana, India). Sodium poly(styrene sulfonate, M.W. 500,000 Powder) (SPS) was obtained from Alfa Aesar (Ward Hill, MA, USA). Acrylic acid (AA) was procured from Acros (Carlsbad, CA, USA) and *N*′,*N*′-methylene bisacrylamide (MBA) was obtained from Alfa Aesar (Lancashire, UK), respectively. Ammonium peroxodisulfate (APS) was acquired from Showa (Tokyo, Japan). All other chemicals and solvents were of analytical reagent grade.

### 3.2. Synthesis of SPSPAA Hydrogels

Various concentrations of SPS, AA, and MBA were cross-lined using a free radical co-polymerization technique for the preparation of sodium poly(styrene sulfonate-co-poly acrylic acid) (SPSPAA) hydrogels as shown in Table 3. SPS was dissolved in a specific amount of deionized distilled water. Similarly, APS was dissolved in a required quantity of distilled water, whereas MBA was dissolved in a mixture of ethanol and water at 50 °C under constant stirring. APS was added slowly into the SPS solution, followed by drop-wise addition of AA. After a few minutes of mixing, MBA was added into the mixture. The mixture was continuously stirred until a translucent solution was formed. Then, the clear solution was poured into glass tubes and kept in a water bath at 55 °C for the initial 2 h and then the temperature was enhanced up to 65 °C for the next 20 h. The developed gel was cut into 8 mm discs. A mixture of water and ethanol (50:50) was used for washing the prepared gel discs to remove any attached unreacted contents. The discs were dried initially at room temperature for 24 h and then placed in a vacuum oven at 40 °C until complete dryness. The developed hydrogel discs were assessed for further studies.

### 3.3. Sol-Gel Analysis

A sol-gel fraction was performed for all formulations of SPSPAA hydrogels to know the sol and gel fraction of the developed system. Gel is the gelling, while sol is the soluble, unreacted part of hydrogels. A Soxhlet extraction process was carried out for analysis in such a way that a hydrogel disc (Z_1_) of precise weight of each formulation was placed in distilled water at 85 °C for 12 h. After that, the extracted hydrogel disc (Z_2_) was kept in an oven at 40 °C until the disc dried completely [40]. Equation (1) and (2) were used for analysis of the sol-gel fraction.

(1) $$\mathrm{Sol}\;\mathrm{fraction}\;\%\;=\frac{Z_{1}-Z_{2}}{Z_{2}}\;\times \;100$$

where, Z_1_ = initial weight of hydrogels disc, and Z_2_ = final weight of hydrogels disc.

(2) Gel fraction=100−Sol fraction 

### 3.4. Swelling Studies

Two different pHs (1.2 and 7.4) were used as swelling media for investigating dynamic swelling of all SPSPAA hydrogel formulations. Primarily, the weighed hydrogels were placed in respective buffer medium at 37 °C. After a specific time interval, hydrogels were removed, blotted off carefully to get rid of any adhered droplets of liquid on the surface, and weighed again on an electronic weighing balance. This process was continued until a constant equilibrium weight was achieved [41]. The given equation was used for calculating dynamic and equilibrium swelling.

(3) $$\left(q\right)=\frac{{\;A}_{2}}{{\;A}_{1}}\;\;$$

where q = dynamic swelling, A_1_ = initial weight before swelling, and A_2_ = final weight after swelling at time t.

### 3.5. Drug Loading

KT 1% solution was used for drug loading. Weighed, dried hydrogels were immersed in drug solution at 25 °C. Phosphate buffer 7.4 was used as solvent for dissolving KT. The immersed, fabricated hydrogels in drug solution were kept until a constant weight was achieved. The drug-loaded hydrogels were placed in a vacuum oven at 40 °C until they dried completely [42,43].

Quantification of loaded drug was conducted by using the dry weight method. In this method, the quantity of loaded drug was analyzed by taking the difference of the weight of the developed hydrogels before and after immersion in the drug solution [43]. This process was repeated three times. The loaded hydrogels were assessed for further studies. The given equation was used for estimation of the amount of drug loaded by the developed hydrogels.

Amount of Drug loaded = W_LD_−W_ULD_ (4)

where W_LD_ = weight of dried, loaded disc of hydrogel, and W_ULD_ = weight of dried unloaded disc of hydrogel.

### 3.6. In-Vitro Drug Release and Kinetics Modeling

An in-vitro drug release study of different formulations of SPSPAA hydrogels was carried out at both acidic and basic medium (pH 1.2 and 7.4) respectively. USP dissolution apparatus II (Sr8plus Dissolution Test Station) was used for the dissolution of drug-loaded hydrogel formulations. The drug-loaded hydrogels were added to 900 mL media of the respective medium at 37 °C and 50 rpm. The samples were taken after regular intervals of time and fresh medium of the same quantity was added to maintain the sink condition. The drug release concentration was analyzed by using a UV–vis-spectrophotometer (U-5100, 3J2-0014, Tokyo, Japan) at the λ max value of 280 nm [31]. The absorbance of samples was taken three times. The Korsymer‒Peppas equation was used for evaluation of drug release data from developed hydrogels’ network [44].

Mt/M∞ = kt^n^ (5)

### 3.7. Thermal Analysis

Thermal analysis of SPS and the hydrogel sample was done on thermal analysis systems: (1) thermogravimetric analysis (TGA) (Simultaneous Thermal Analyzer STA 8000, Perkin Elmer, Waltham, MA, USA) and (2) differential scanning calorimetry (DSC) (PerkinElmer DSC 4000, Waltham, MA, USA). For TGA analysis, samples were heated between 20 and 600 °C at a constant flow of nitrogen. Similarly, for DSC analysis, nitrogen flow was maintained the same throughout the study with a heating rate of 20 °C/min up to 400 °C [45].

### 3.8. Powder X-ray Diffraction (PXRD) Analysis

Powder X-ray diffraction (XRD-6000 Shimadz, Tokyo, Japan) was conducted for nature analysis of SPSPAA hydrogels. The angle of diffraction was varied from 10° to 60° at a rate of 2° 2θ/min [45].

### 3.9. Fourier Transform Infrared Spectroscopy (FTIR)

FTIR was carried out for SPS, AA, unloaded SPSPAA hydrogels, KT, and drug-loaded SPSPAA hydrogels. All the samples were carefully crushed in a pestle and mortar to the desired size and then Nicolet 380 FTIR (Thermo Fisher Scientific, Ishioka, Japan) was employed for analysis in the range of 4000–500 cm^−1^ [46].

### 3.10. Scanning Electron Microscopy (SEM)

SEM (JSM-5300 model, Jeol Ltd., Tokyo, Japan) was used for analysis of the surface morphology of the developed hydrogels. Scanning of fabricated hydrogels was performed at various magnifications [47].

## 4. Conclusions

SPSPAA hydrogels were prepared successfully by using a free radical co-polymerization technique. Dynamic swelling and percent drug release studies indicated pH-dependent behavior as both swelling and drug release was found higher at pH 7.4 compared to pH 1.2 due to the deprotonation of functional groups of both polymer and monomer. TGA and DSC revealed higher thermal stability of fabricated hydrogels as compared to pure unreacted polymer. PXRD demonstrated that the developed network of the hydrogel was less crystalline than polymer as the intensity of broad peaks of polymer was reduced in the developed system due to the cross-linking of the polymer with monomer. FTIR studies indicated the successful reaction of polymer with monomer and monomer overlapping on the backbone of the polymer. SEM indicated a rough surface with few pores of the developed hydrogels. Conclusively, SPSPAA could be considered as a polymer carrier for controlled delivery of NSAIDs to overcome the limitation concerned with their repeated administration.

## Figures and Tables

**Table 1:** Sol-gel analysis, dynamic swelling, and drug loading of sodium poly(styrene sulfonate-co-poly acrylic acid (SPSPAA) hydrogels.

Formulation	Sol Fraction	Gel Fraction	Dynamic Swelling Up to 36 h		Drug Loaded
Code	(%)	(%)	pH 1.2	pH 7.4	(mg)/400 mg of Dry Gels
PSAF-1	14.88	85.12	3.16 ± 0.12	14.39 ± 0.24	170 ± 1.12
PSAF-2	11.10	88.90	3.18 ± 0.14	16.33 ± 0.38	186 ± 0.92
PSAF-3	9.24	90.76	3.24 ± 0.23	17.17 ± 0.21	198 ± 0.98
PSAF-4	16.53	83.47	3.08 ± 0.08	9.35 ± 0.33	155 ± 1.06
PSAF-5	14.88	85.12	3.16 ± 0.12	14.39 ± 0.24	170 ± 1.12
PSAF-6	10.72	89.28	3.28 ± 0.38	15.98 ± 0.43	220 ± 1.22
PSAF-7	14.88	85.12	3.16 ± 0.12	14.39 ± 0.24	170 ± 1.12
PSAF-8	13.12	86.88	3.04 ± 0.27	11.16 ± 0.37	110 ± 1.18
PSAF-9	10.59	89.41	2.80 ± 0.24	10.32 ± 0.32	94 ± 1.14

**Table 2:** Kinetic modeling release of KT from SPSPAA hydrogels.

Formulation	Korsmeyer‒Peppas Model	
Code	r^2^	N
PSAF-1	0.8949	0.3396
PSAF-2	0.9219	0.3684
PSAF-3	0.9724	0.3927
PSAF-4	0.9743	0.5299
PSAF-5	0.8949	0.3396
PSAF-6	0.8832	0.3465
PSAF-7	0.8949	0.3396
PSAF-8	0.9370	0.3818
PSAF-9	0.9829	0.4420

**Table 3:** Chemical composition of SPSPAA hydrogels.

Formulation	Polymer	Monomer	Initiator	Cross-Linker
	(SPS)	(AA)	(APS)	(MBA)
Code	g/100 g	g/100 g	g/100 g	g/100 g
PSAF-1	0.50	25	0.5	0.50
PSAF-2	0.75	25	0.5	0.50
PSAF-3	1.00	25	0.5	0.50
PSAF-4	0.50	20	0.5	0.50
PSAF-5	0.50	25	0.5	0.50
PSAF-6	0.50	30	0.5	0.50
PSAF-7	0.50	25	0.5	0.50
PSAF-8	0.50	25	0.5	0.75
PSAF-9	0.50	25	0.5	1.00

## Data Availability

Not applicable.
